# Stearoyl-CoA Desaturase-1 Attenuates the High Shear Force Damage Effect on Human MG63 Osteosarcoma Cells

**DOI:** 10.3390/ijms21134720

**Published:** 2020-07-02

**Authors:** Kuo-Chin Huang, Po-Yao Chuang, Rong-Ze Hsieh, Cheng-Nan Chen, Shun-Fu Chang, Yu-Ping Su

**Affiliations:** 1 Department of Orthopaedics, Chiayi Chang Gung Memorial Hospital, Chiayi 613, Taiwan; kc2672@adm.cgmh.org.tw (K.-C.H.); b9102072@cgmh.org.tw (P.-Y.C.); 2 College of Medicine, Chang Gung University, Taoyuan 333, Taiwan; 3 Department of Food Science, National Chiayi University, Chiayi 600, Taiwan; 4 Graduate Institute of Clinical Medical Sciences, College of Medicine, Chang Gung University, Taoyuan 333, Taiwan; atmw113106@gmail.com; 5 Department of Biochemical Science and Technology, National Chiayi University, Chiayi 600, Taiwan; cnchen@mail.ncyu.edu.tw; 6 Department of Medical Research and Development, Chiayi Chang Gung Memorial Hospital, Chiayi 613, Taiwan; 7 Department of Orthopaedics and Traumatology, Veterans General Hospital, Taipei 112, Taiwan; 8 Department of Surgery, School of Medicine, National Yang-Ming University, Taipei 112, Taiwan

**Keywords:** high shear force, peroxisome proliferator-activated receptor δ, osteosarcoma, Smad1/5, Stearoyl-CoA desaturase-1

## Abstract

Mechanical regulation is known as an important regulator in cancer progression and malignancy. High shear force has been found to inhibit the cell cycle progression and result in cell death in various cancer cells. Stearoyl-CoA desaturase (SCD)-1, one of the important lipogenic enzymes, has recently been indicated as a potential pharmaceutical target in cancer therapy. In this study, we determined whether the cell fate control of shear force stimulation is through regulating the SCD-1 expression in cancer cells. Human MG63 osteosarcoma cells were used in this study. 2 and 20 dynes/cm^2^ shear forces were defined as low and high intensities, respectively. A SCD-1 upregulation in human MG63 osteosarcoma cells under 20, but not 2, dynes/cm^2^ shear force stimulation was shown, and this induction was regulated by Smad1/5 and peroxisome proliferator-activated receptor δ (PPARδ) signaling. Moreover, gene knockdown of PPARδ and SCD-1 in human MG63 osteosarcoma cells attenuated the differentiation inhibition and resulted in much more cell death of high shear force initiation. The present study finds a possible auto-protective role of SCD-1 upregulation in high shear force-damaged human MG63 osteosarcoma cells. However, its detailed regulation in the cancer fate decision of high shear force should be further examined.

## 1. Introduction

Mechanical regulation of cancer has been evidenced as having an important role in progression and malignance [1]. In addition to the stimulation of biochemical stimulators, including cytokines and chemokines, the vast change of the mechanical microenvironment in cancer tissues is also a cause of abnormal growth and aggressive property of cancer cells [2,3]. It has been shown that the extracellular matrix reconstructions, including the alterations of composition, viscoelasticity (microrheology) and fibrous structure and porosity, usually lead to the stiffening of cancer tissues [4,5]. Accumulating data has further suggested a possibility of considering matrix stiffening as a predictor of cancer metastasis and progression [6,7]. Liquid flow in blood, lymphoid and interstitial fluids plays an important mechanical role in controlling the physiological and pathophysiological development. During the cancer progression, the stiffening of the cancer matrix gradually increases the intratumoral pressure and hence leads to the increased and disturbed blood and interstitial flows with a flux from the cancer tissue to the surrounding healthy stroma [8,9]. It has been discovered that the different intensities of shear forces resulted from these blood and interstitial flows are correlated with the growth, survival and metastasis of different types of cancer cells, including breast, oral and renal carcinoma, glioma, and osteo- and chondro-sarcoma [9]. Moreover, further mechanism studies have elucidated that Smad1/5, matrix metalloproteases, transforming growth factor-β and chemokine signaling might be regulated by shear forces to affect the cancer cell fates [9].

Tremendous fatty acid (FA) *de novo* synthesis has been found in cancer cells to support their intensive demand for unregulated growth and aggressiveness [10,11]. FAs in mammalian cells involve saturated fatty acids (SFAs) and monounsaturated fatty acids (MUFAs), which are the basic constituents of phospholipids, di/triacylglycerols and cholesteryl esters, and hence could be used in the cell membrane construction, energy store and signal transduction in cells [10,12]. These features could also just satisfy the higher proliferation rate and energy consumption of cancer cells. Stearoyl-CoA desaturase (SCD)-1 is an endoplasmic reticulum-associated enzyme. Its enzymatic activity could induce a conversion of SFA to MUFA [13,14]. Accumulating basic and clinical studies have found a lower serum ratio of SFA to MUFA in patients with breast, prostate, colorectal, liver and pancreatic cancers compared to healthy individuals. The serum SFA/MUFA ratio has hence been suggested to be a potential diagnostic tool in patients with these cancers [15,16,17]. SCD-1 inhibition has also hence been proposed as a great pharmaceutical target for anticancer drug development [10]. SCD-1 level in cells is mainly regulated by a transcription process. Previous studies have indicated that sterol regulatory element binding protein-1 might be the primary transcription factor to regulate SCD-1 expressions. However, accumulating data has further found that many others transcription factors, including peroxisome proliferator-activated receptors (PPARs), neurofibromin 1, CCAAT/enhancer binding protein-α and liver X receptor, could also regulate SCD-1 expression in cancer cells in response to different stimulators or inducers [10,18,19,20]. The transcription regulation of SCD-1 in cancer cells is complex and is a context-dependent event. Although more researchers have started to explore the pharmaceutical role of SCD-1 in cancer therapy, it still needs more efforts to elucidate the precise regulatory mechanism in its expression level in cancer cells under different stimulations.

Our previous studies demonstrated that high shear force could result in the cell cycle arrest and cell death in multiple types of cancer cells through Smad1/5 signaling [21,22]. In the present study, we further investigated the possible role of SCD-1 in shear force effect on human MG63 osteosarcoma cells. We found that 20, but not 2, dynes/cm^2^ shear force surprisingly increases the SCD-1 levels in human MG63 osteosarcoma cells through the activation of Smad1/5 signaling and PPARδ transcriptional factor. Moreover, this SCD-1 induction could contribute to cell fate regulation in human MG63 osteosarcoma cells.

## 2. Results

### 2.1. 20 dynes/cm^2^ Shear Force Induces SCD-1 Protein and mRNA Expressions in Human MG63 Osteosarcoma Cells

**Figure 1:**
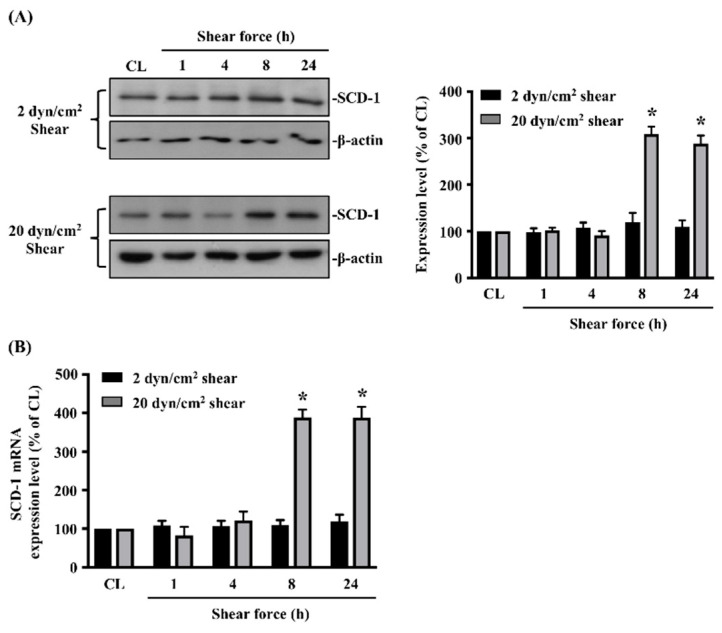
20 dynes/cm^2^ shear force induces stearoyl-CoA desaturase (SCD)-1 protein and messenger RNA (mRNA) expressions in human MG63 osteosarcoma cells. (**A**,**B**) Cells were kept as the controls (CL) or stimulated with 2 or 20 dynes/cm^2^ shear force for 1, 4, 8 and 24 h, and their protein (**A**) and mRNA (**B**) expressions of SCD-1 were analyzed by Western blot and real-time quantitative polymerase chain reaction (PCR), respectively. Results in (**A**) are representative of three independent experiments with similar results. Statistical data in (**A**,**B**) are mean ± standard error of the mean (SEM) from three independent experiments. Protein (**A**) and mRNA (**B**) levels were normalized to β-actin and glyceraldehyde-3-phosphate dehydrogenase (GAPDH), respectively. * *p* < 0.05 versus untreated control cells (CL).

Cells were kept as the controls or stimulated with 2 or 20 dynes/cm^2^ shear force for 1, 4, 8 and 24 h, and their protein and mRNA expressions of SCD-1 were analyzed. It was shown that 20 dynes/cm^2^ shear force significantly induces SCD-1 protein (Figure 1A and Supplementary Figure S1) and mRNA (Figure 1B) expressions in human MG63 osteosarcoma cells after 8 and 24 h of treatment, which is approximately 3~4 times that of the untreated controls. 2 dynes/cm^2^ shear force had no effect on SCD-1 protein (Figure 1A and Supplementary Figure S1) and mRNA (Figure 1B) expressions.

### 2.2. Smad1/5 Signaling Regulates 20 dynes/cm^2^ Shear Force-Induced SCD-1 Expression in Human MG63 Osteosarcoma Cells

**Figure 2:**
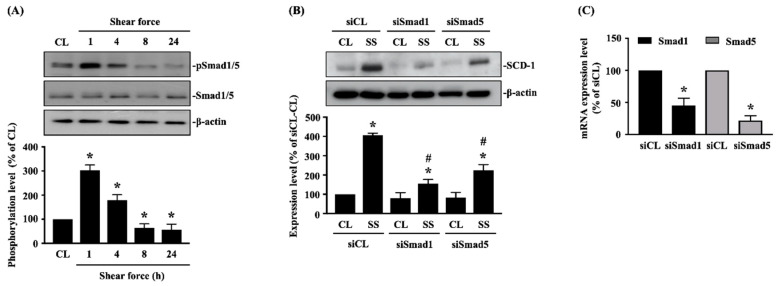
Smad1/5 signaling regulates 20 dynes/cm^2^ shear force-induced SCD-1 expression in human MG63 osteosarcoma cells. (**A**) Cells were kept as the controls (CL) or stimulated with 20 dynes/cm^2^ shear force for 1, 4, 8 and 24 h, and their Smad1/5 phosphorylation was analyzed by Western blot. (**B**,**C**) Cells were transfected with *control*-, *Smad1*-, or *Smad5*-specific siRNA (siCL, siSmad1, siSmad5) and then kept as the controls (CL) or stimulated with 20 dynes/cm^2^ shear force (SS) for 8 h. (**B**) The SCD-1 protein expression was analyzed by Western blot. (**C**) The *Smad1* or *Smad5* gene expressions were analyzed by real-time quantitative PCR. Results in (**A**,**B**) are representative of three independent experiments with similar results. Statistical data in (**A**–**C**) are mean ± SEM from three independent experiments. Protein phosphorylation and expression (**A**,**B**) and mRNA expression (**C**) levels were normalized to Smad1/5/β-actin and GAPDH, respectively. * *p* < 0.05 versus untreated control cells (CL), siCL-CL, or siCL. # *p* < 0.05 versus siCL-SS-treated cells.

The evidence from others and us showed that Smad1/5 signaling is one of the shear-stimulated mechanotransductions [21,22]. Thus, we examined whether Smad1/5 signaling also affects 20 dynes/cm^2^ shear force-induced SCD-1 expression in human MG63 osteosarcoma cells. Cells were kept as the controls or stimulated with 20 dynes/cm^2^ shear force for 1, 4, 8 and 24 h, and their Smad1/5 phosphorylation was analyzed. It was shown that 20 dynes/cm^2^ shear force significantly induces Smad1/5 phosphorylation in human MG63 osteosarcoma cells within 1 h of treatment, and this induction was returned to the basal level after 4 h of treatment (Figure 2A and Supplementary Figure S2). Cells were transfected with *Smad1*- or *Smad5*-specific small interfering RNA (siRNA) to knockdown the corresponding gene expressions and then kept as the controls or stimulated with 20 dynes/cm^2^ shear force for 8 h. *Smad1* and *Smad5* gene knockdown significantly inhibited SCD-1 protein expression of 20 dynes/cm^2^ shear force induction in MG63 osteosarcoma cells (Figure 2B and Supplementary Figure S2). *Smad1*- and *Smad5*-specific siRNA induced a ~50–80% reduction of corresponding gene expressions (Figure 2C).

### 2.3. Smad1/5 Increase PPARδ Expression and Transcription Activation to Regulate SCD-1 Upregulation of 20 dynes/cm^2^ Shear Force Stimulation in Human MG63 Osteosarcoma Cells

**Figure 3:**
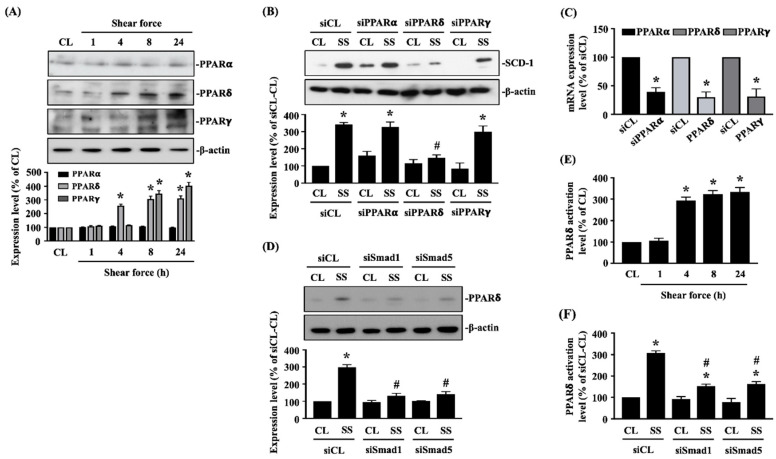
Smad1/5 increase peroxisome proliferator-activated receptor δ (PPARδ) expression and transcription activation to regulate SCD-1 upregulation of 20 dynes/cm^2^ shear force stimulation in human MG63 osteosarcoma cells. (**A**,**E**) Cells were kept as the controls (CL) or stimulated with 20 dynes/cm^2^ shear force for 1, 4, 8 and 24 h, and their PPARs expressions (**A**) and PPARδ transcription activity (**E**) were analyzed by Western blot and transcription factor ELISA assay, respectively. (**B**–**D**,**F**) Cells were transfected with *control*- *PPARα*-, *PPARδ*-, or *PPARγ*-specific siRNA (siCL, siPPARα, siPPARδ, siPPARγ) (**B**,**C**) or *Smad1*- or *Smad5*-specific siRNA (siSmad1, siSmad5) (**D**,**F**) and then kept as the controls (CL) or stimulated with 20 dynes/cm^2^ shear force (SS) for 8 h. (**B**) The SCD-1 expression was analyzed by Western blot. (**C**) The *Smad1* or *Smad5* gene expressions were analyzed by real-time quantitative PCR. (**D**,**F**) The PPARδ expression (**D**) and transcription activity (**F**) were analyzed by Western blot and transcription factor ELISA assay, respectively. Results in (**A**,**B**,**D**) are representative of three independent experiments with similar results. Statistical data in (**A**–**F**) are mean ± SEM from three independent experiments. Protein (**A**,**B**,**D**) and mRNA (**C**) expression levels were normalized to β-actin and GAPDH, respectively. * *p* < 0.05 versus untreated control cells (CL), siCL-CL, or siCL. # *p* < 0.05 versus siCL-SS-treated cells.

PPARs have been implicated in regulating *SCD-1* gene expression [10]. Thus, cells were kept as the controls or stimulated with 20 dynes/cm^2^ shear force for 1, 4, 8 and 24 h, and their PPARs expressions were analyzed. It was shown that 20 dynes/cm^2^ shear force significantly induces PPARδ and PPARγ protein expressions after 4, 8 and 24 h of stimulations in human MG63 osteosarcoma cells compared to the untreated controls (Figure 3A and Supplementary Figure S3). 20 dynes/cm^2^ shear force did not change the PPARα expression. Cells were transfected with *PPARα*-, *PPARδ*- or *PPARγ*-specific siRNA to knockdown the corresponding gene expressions and then kept as the controls or stimulated with 20 dynes/cm^2^ shear force for 8 h. It was shown that only *PPARδ* gene knockdown inhibits 20 dynes/cm^2^ shear force-induced SCD-1 protein expression in human MG63 osteosarcoma cells (Figure 3B and Supplementary Figure S3). *PPARα*-, *PPARδ*- or *PPARγ*-specific siRNA induced a ~60–70% reduction of corresponding gene expressions (Figure 3C). Cells were further transfected with *Smad1*- or *Smad5*-specific siRNA to knockdown the corresponding gene expressions and then kept as the controls or stimulated with 20 dynes/cm^2^ shear force for 8 h. *Smad1* or *Smad5* gene knockdown in MG63 osteosarcoma cells blocked the PPARδ protein expression of 20 dynes/cm^2^ shear force induction (Figure 3D and Supplementary Figure S3). By using the PPARδ transcription factor enzyme-linked immunosorbent assay (ELISA), as expected, 20 dynes/cm^2^ shear force significantly increased PPARδ transcription activity in a time-dependent manner in human MG63 osteosarcoma cells compared to the untreated controls (Figure 3E). *Smad1* or *Smad5* gene knockdown in human MG63 osteosarcoma cells also blocked the PPARδ transcription activation (Figure 3F).

### 2.4. SCD-1 Regulates 20 dynes/cm^2^ Shear Force-Inhibited Differentiation Marker Expressions in MG63 Osteosarcoma Cells

**Figure 4:**
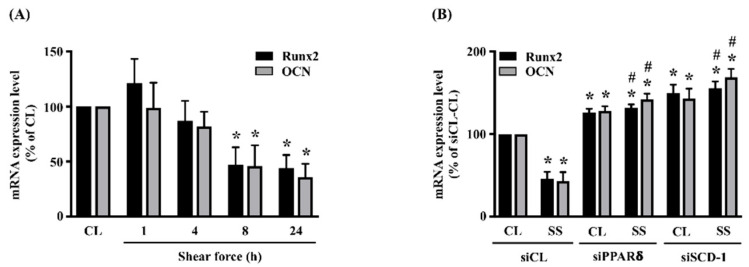
SCD-1 regulates 20 dynes/cm^2^ shear force-inhibited differentiation marker expressions in MG63 osteosarcoma cells. (**A**) Cells were kept as the controls (CL) or stimulated with 20 dynes/cm^2^ shear force for 1, 4, 8 and 24 h, and their *Runx2* and *OCN* mRNA expressions were analyzed by real-time quantitative PCR. (**B**) Cells were transfected with *control*-, *PPARδ*-, or *SCD-1*-specific siRNA (siCL, siPPARδ, siSCD-1) and then kept as the controls (CL) or stimulated with 20 dynes/cm^2^ shear force (SS) for 8 h. The *Runx2* and *OCN* mRNA expressions were analyzed by real-time quantitative PCR and normalized to GAPDH. Statistical data in (**A**,**B**) are mean ± SEM from three independent experiments. * *p* < 0.05 versus untreated control cells (CL) or siCL-CL. # *p* < 0.05 versus siCL-SS-treated cells.

To further explore the role of SCD-1 upregulation in MG63 osteosarcoma cells under 20 dynes/cm^2^ shear force stimulation, cells were kept as the controls or stimulated with 20 dynes/cm^2^ shear force for 1, 4, 8 and 24 h, and their differentiation markers, i.e., *Runx2* and *osteocalcin* (*OCN*), were analyzed. It was shown that 20 dynes/cm^2^ shear force inhibits the mRNA expressions of *Runx2* and *OCN* in human MG63 osteosarcoma cells after 8 and 24 h of stimulations (Figure 4A). Moreover, cells transfected with *PPARδ*- or *SCD-1*-specific siRNA to knockdown the corresponding gene expression significantly recovered the inhibitory effect of 20 dynes/cm^2^ shear force on *Runx2* and *OCN* mRNA expressions in human MG63 osteosarcoma cells (Figure 4B). Interestingly, gene knockdown of *PPARδ* or *SCD-1* seemed to partially increases the endogenous *Runx2* and *OCN* mRNA expressions in MG63 osteosarcoma cells (Figure 4B).

### 2.5. SCD-1 Regulates 20 dynes/cm^2^ Shear Force-Stimulated Cell Death in Human MG63 Osteosarcoma Cells

**Figure 5:**
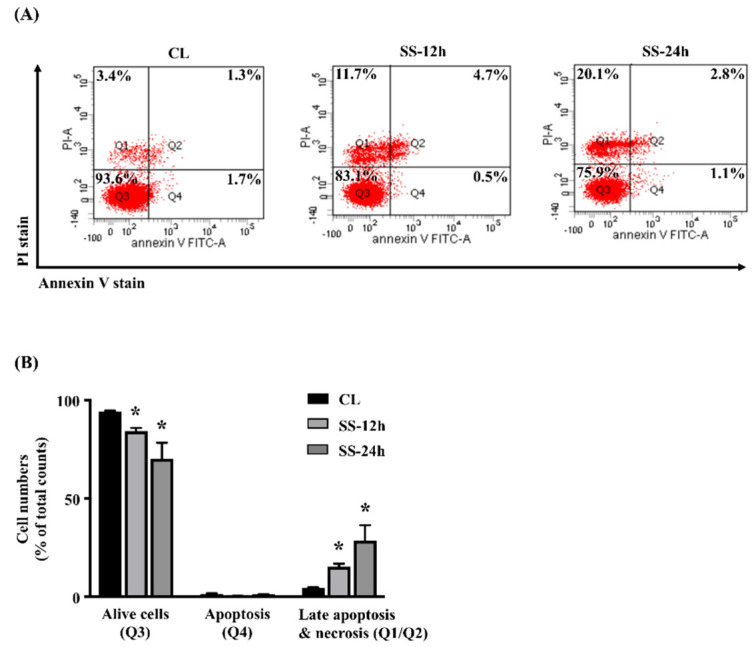
20 dynes/cm^2^ shear force results in the cell death of human MG63 osteosarcoma cells. (**A**) Cells were kept as the controls (CL) or stimulated with 20 dynes/cm^2^ shear force (SS) for 12 and 24 h, and their cell death were analyzed by propidium iodide (PI)/Annexin V double stain and flow cytometry. Q1 + Q2 = late apoptotic and necrotic cells, Q3 = living cells, Q4 = apoptotic cells. (**B**) quantification of apoptotic and necrotic cells. Statistical data are mean ± SEM from three independent experiments. * *p* < 0.05 versus untreated control cells (CL).

**Figure 6:**
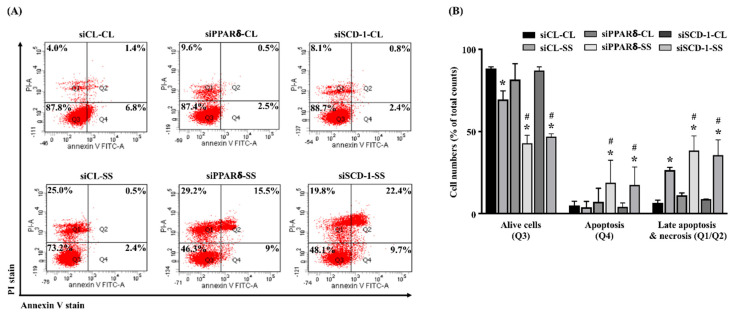
SCD-1 regulates 20 dynes/cm^2^ shear force-stimulated cell death in human MG63 osteosarcoma cells. (**A**) Cells were transfected with *control*-, *PPARδ*-, or *SCD-1*-specific siRNA (siCL, siPPARδ, siSCD-1) and then kept as the controls (CL) or stimulated with 20 dynes/cm^2^ shear force (SS) for 24 h, and their cell death was analyzed by PI/Annexin V double stain and flow cytometry. Q1 + Q2 = late apoptotic and necrotic cells, Q3 = living cells, Q4 = apoptotic cells. (**B**) quantification of apoptotic and necrotic cells. Statistical data are mean ± SEM from three independent experiments. * *p* < 0.05 versus siCL-CL. # *p* < 0.05 versus siCL-SS-treated cells.

Next, we determined whether SCD-1 upregulation in MG63 osteosarcoma cells under 20 dynes/cm^2^ shear force stimulation also controls the cell death. Cells were kept as the controls or stimulated with 20 dynes/cm^2^ shear force for 12 and 24 h, and their cell apoptosis/necrosis were analyzed. It was shown that 20 dynes/cm^2^ shear force significantly results in a decrease in the live cells and an increase in the late apoptotic and necrotic cells of human MG63 osteosarcoma cells in a time-dependent manner compared to the control cells (Figure 5A,B). Moreover, interesting results showed that cells transfected with *PPARδ*- or *SCD-1*-specific siRNA to knockdown the corresponding gene expression enhance the cell death effect of 20 dynes/cm^2^ shear force stimulation more in human MG63 osteosarcoma cells (Figure 6A,B).

## 3. Discussion

**Figure 7:**
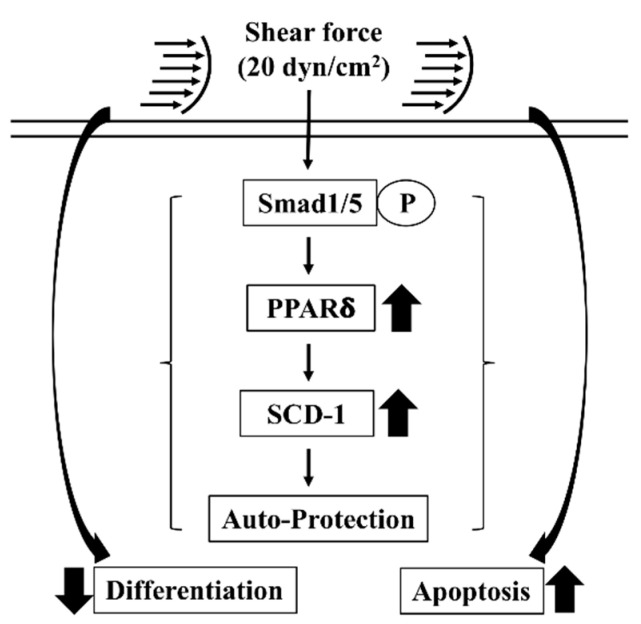
Schematic representation of a possible mechanism affecting 20 dynes/cm^2^ shear force-stimulated SCD-1 expression and consequent differentiation and cell death regulations in human MG63 osteosarcoma cells.

Fluid shear forces from irregular blood and interstitial flows in the cancer mechanical microenvironment have been found to be an important mechanical regulator in regulating the cancer cell fates [8,9]. Previous studies from others and us demonstrated that bone morphogenetic protein-independent Smad1/5 signaling activation of shear force stimulation could result in the cell cycle arrest, differentiation inhibition and cell death of different types of cancer cells [21,22]. The proposed study was carried out on these bases and further found the correlation between lipid metabolism and shear force regulation of cancer cells, which elucidated a possible auto-protective role of SCD-1 upregulation in high shear force (20 dynes/cm^2^)-stimulated cell death of osteosarcoma (summarized in Figure 7). The systematic experiments demonstrated that (i) 20, but not 2, dynes/cm^2^ shear force increases SCD-1 protein and mRNA expression levels in human MG63 osteosarcoma cells, (ii) Smad1/5 signaling and PPARδ transcription factor mediates SCD-1 upregulation in human MG63 osteosarcoma cells under high shear force stimulation and (iii) gene knockdown of PPARδ and SCD-1 in human MG63 osteosarcoma cells attenuates the differentiation inhibition and promotes much more cell death of high shear force initiation. Our findings elucidate an interesting role of SCD-1 upregulation in human osteosarcoma while the cells are exposed to high shear force and suggest that this SCD-1 upregulation might contribute a complicated regulatory mechanism to affect the cancer cell survival.

Because of the microenvironmental restrictions, the tumor vascular system is always poorly developed and subsequently results in the poor supply of nutrients and oxygen [23,24]. In order to cope with uncontrollable growth and proliferation, cancer cells should synthesize the lipids, including SFA and MUFA, *de novo* to satisfy the vast demands of energy and cell structural components [10,11,12]. In that case, the request and importance of elevated and activated lipogenic enzymes, including SCD-1, in the progression of cancer survival, metastasis and malignancy have hence been demonstrated [11]. Moreover, a possibility has been further proposed that SCD-1 activity inhibition could become a potential target of anti-cancer therapy [10]. The data from our previous and present studies demonstrated that high shear force could inhibit differentiation and initiate cell death of the cancer cells, including the human MG63 osteosarcoma, through Smad1/5 signaling [21,22]. The present study further found a surprising upregulation of SCD-1 level in human MG63 osteosarcoma cells in response to this destructed high shear force. Moreover, while the SCD-1 gene was knocked down in human MG63 osteosarcoma cells, the differentiation inhibition and cell death of high shear force stimulation were significantly attenuated and more promoted, respectively. Although our results could not completely provide the precise explanation yet, we reasonably suggested that SCD-1 upregulation in high shear force-damaged MG63 osteosarcoma cells might be an auto-protective mechanism. This suggestion could be supported by a perspective that differentiation promotion might be one novel anti-osteosarcoma therapeutic strategy [25] and by the increasing data finding a positive correlation between SCD-1 activity inhibition and cancer cell death [26,27,28].

Differentiation promotion therapy has been proposed since the 1970s and has been applied in clinical treatment of acute promyelocytic leukemia [25,29]. However, because of the complexity of differentiation signaling in various cancer types, the clinical application and efficiency of differentiation promotion therapy in other solid cancers has not been evidenced. Osteosarcoma is one of the poorly differentiated cancers and its current clinical treatment methods encounter many difficulties [30,31,32]. Recently, accumulating data has suggested that the differentiation promotion therapy with differentiation agents, including PPARγ agonists, in osteosarcoma might be a potential theranostic strategy and has been extensively examined [25,33]. Our results about the inhibition of SCD-1 and its transcription regulator, i.e., PPARδ, in MG63 osteosarcoma cells could recover the high shear force-inhibited gene expression of differentiation markers and might provide a new hint if SCD-1 activity and expression regulation could also be applied in differentiation promotion therapy of osteosarcoma.

PPARδ is ubiquitously expressed in most tissues but its function is the least well-characterized among the three PPAR members [34]. In the bone biology, both PPARδ and PPARγ, not PPARα, have been indicated as the major mediators for lipogenesis and lipid metabolism. For the SCD-1 transcription regulation, PPARα is the only well-defined transcription factor to mediate the SCD-1 expression [11]. However, in the present results, only PPARδ and PPARγ, but not PPARα, expressions were increased in MG63 osteosarcoma cells in response to high shear force stimulation, and further results found that only PPARδ is involved in mediating SCD-1 upregulation of high shear force stimulation. A previous study found a positive correlation between the SCD-1 activity and PPARδ expression, but their interacting mechanism has not been clearly examined [35]. In the vascular studies, it has been demonstrated that PPARδ could be activated by shear force and subsequently affect the endothelial cell functions and smooth muscle cell phenotypic modulation [36,37]. Combined with these studies, we suggested that transcription regulation of SCD-1 is also in a context-dependent manner and PPARδ could be another transcription mediator in osteosarcoma cells under high shear force stimulation.

The measurement of exact intratumoral shear force intensity initiated by interstitial flow is difficult. Generally, the flow rate in the tumor periphery (~0.1–4.0 µm/s) is higher than that in the center (~0 µm/s), which is a result of the intratumoral gradient pressure [21]. Moreover, previous studies have indicated that the in vivo shear loading on bone cells might be 8–30 dynes/cm^2^ [21]. Thus, combined with these previous findings, the present study defined 2 and 20 dynes/cm^2^ shear intensity as low and high shear force respectively, to investigate the shear effect on SCD-1 regulation in osteosarcoma cells and subsequent cell fate control. However, the limitations in our findings, including using a single cell line only and no data on the SFA/MUFA assays, and eventually in vivo evidence, still need to be further addressed in the future.

The present study surprisingly found an induction of SCD-1 levels in human osteosarcoma cells under high shear force stimulation through Smad1/5-PPARδ signaling, which consequently affects the osteosarcoma survival (differentiation and cell death). These results elucidate an auto-protection possibility of SCD-1 upregulation in the osteosarcoma cells in response to high shear force damage. However, the more detailed and precise role of SCD-1 upregulation should be further investigated in various osteosarcoma cells and in vivo experimental designs.

## 4. Materials and Methods

### 4.1. Materials

Smad1/5/9 antibody (1:1000) was obtained from Abcam (ab80255, Cambridge, MA, USA). All other antibodies, including SCD-1 (#2794, 1:1000), phospho-Smad1/5/9 (#13820, 1:1000) and β-actin (#4970, 1:5000), were obtained from Cell Signaling Technology (Beverly, MA, USA). The gene-specific siRNAs, including *control* (AM4615), *Smad1* (HSS106248), *Smad5* (HSS106259), *PPARα* (s10882), *PPARδ* (s10885) and *PPARγ* (s10888), were obtained from Thermo (Waltham, MA, USA). The specific siRNA for *SCD-1* (SG00327274) were obtained from Sigma-Aldrich (St Louis, MO, USA). All other chemicals of reagent grade were obtained from Sigma-Aldrich (St Louis, MO, USA).

### 4.2. Cell Culture and Shear Force Experiment

Human MG63 osteosarcoma cells were purchased from American Type Culture Collection (ATCC) cell bank (67th passage, Rockville, MD, USA) and cultured in 10% fetal bovine serum (FBS)/1% antibiotics (penicillin and streptomycin) containing Dulbecco’s modified Eagle’s medium. FBS, antibiotics and medium were purchased from Invitrogen (Carlsbad, CA, USA). MG63 osteosarcoma cells (75th–82nd passages only) were seeded onto the glass slides, pre-coated with type I collagen (30 µg/mL, at 37 °C for 1 h) and then incubated in a 37 °C incubator with 5% CO_2_ for 24 h in the flow-shear experiments, as previously described [21,22]. After that, the slides were further mounted in the parallel-plate flow shear chambers and connected to a perfusion loop system, which were then kept in a temperature/CO_2_-controlled enclosure (37 °C, 5% CO_2_). The shear intensity (*τ*) applied onto the human MG63 osteosarcoma cells was calculated to be 2 and 20 dynes/cm^2^ by the formula: *τ* = 6 *μQ/wh*^2^ (*μ:* viscosity of perfusate, *Q:* flow rate, *w:* width, and *h:* channel height). 2 and 20 dynes/cm^2^ were defined as low and high shear forces, respectively.

### 4.3. Real-Time Quantitative Polymerase Chain Reaction (PCR)

The RNA was isolated from human MG63 osteosarcoma cells by the TRIzol reagent (Invitrogen, Carlsbad, CA, USA)/phenol/chloroform method and converted to cDNA by the SuperScript II Reverse Transcriptase System (Invitrogen, Carlsbad, CA, USA). Briefly, equal concentrations of RNAs were mixed with SuperScript II-Reverse Transcriptase in buffer (0.5 μg/mL oligo-dT primer/10 mM dithiothreitol (DTT) /20 mM Tris-HCl/2.5 mM MgCl2/0.5 mM deoxynucleoside triphosphate mix) and incubated at 42 °C for 50 min. The reaction was finally terminated by adding the RNase H. Real-time quantitative PCR was performed and analyzed in a real-time quantitative PCR machine: ABI StepOnePlus (Applied Biosystems, Foster City, CA, USA), by using the SYBR Green kit (Applied Biosystems, Foster City, CA, USA). Primers are as follows: Smad1 (sense: 5′-TGTGTACTATACGTATGAGCTTTGTGA-3′; antisense: 5′-TAACATCCTGGCGGTGGTA-3′), Smad5 (sense: 5′-TTCGGATGAGTTTTGTCAAGG-3′; antisense: 5′-GGGGTGCTGGTTACATCCT-3′), PPARα (sense: 5′-CCATCGGCGAGGATAGTTCTG-3′; antisense: 5′-TCTACATTCGATGTTCAATGCTCCA-3′), PPAR δ (sense: 5′-AAGGCATCGGGCTTCC ACTA-3′; antisense: 5′-GCACTTCTGGAAGCGGCAGTA-3′), PPARγ (sense: 5′-TGGAATTAGATGA CAGCGACTTGG-3′; antisense: 5′-CTGGAGCAGCTTGGCAAACA-3′), SCD-1 (sense: 5′-CCCAGCC GTCAAAGAGAA-3′; antisense: 5′-CGATGGCGTAACGAAGAAA-3′), Runx2 (sense: 5′-CCGCACG ACAACCGCACCAT-3′; antisense: 5′-CGCTCCGGCCCACAAATCTC-3′), OCN (sense: 5′-CTCACA CTCCTCGCCCTATT-3′; antisense: 5′-TTGGACACAAAGGCTGCAC-3′) and glyceraldehyde-3-phosphate dehydrogenase (GAPDH) (sense:5′-AG AACATCATCCCTGCATCC-3′; antisense: 5′-TTACTCCTTGGAGGCCATGT-3′). The GAPDH expression level was indicated to be an internal control. Real-time quantitative PCR was analyzed in triplicate and by using the 2^−ΔΔ*C*t^ method.

### 4.4. Western Blot

The protein lysates from human MG63 osteosarcoma cells were obtained by adding the lysis buffer (0.1% sodium dodecyl sulfate (SDS)/0.5% sodium deoxycholate/1% NP-40 and a protease/phosphatase inhibitor cocktail). After quantification, equal concentrations of protein lysates (45 µg) were loaded and separated in SDS-polyacrylamide gel electrophoresis (10% running and 4% stacking) and then were transferred onto the nitrocellulose (NC) paper with 0.45 µm pore size. The NC papers were further hybridized and analyzed by adding the indicated primary/secondary antibodies and detected in a Western-Light chemiluminescent detection system (Applied Biosystems, Foster City, CA, USA). β-actin served as a loading control.

### 4.5. Transfection

Human MG63 osteosarcoma cells (80% confluence) were cultured for 24 h and then transfected with the indicated siRNA (40 nM), which could knockdown ~50–80% expression of the corresponding gene compared to the control siRNA-transfected cells. The transfection kit is RNAiMax (Invitrogen, Carlsbad, CA, USA).

### 4.6. PPARδ Transcription Factor Assay

The nuclear extract of human MG63 osteosarcoma cells were isolated by the extract kit (ab113474, Abcam, Cambridge, MA, USA) and the PPARδ transcription activity was analyzed by the PPARδ transcription factor assay kit (ab133106, Abcam, Cambridge, MA, USA). Briefly, the equal concentrations of the nuclear extracts were added into the plate immobilized with a DNA fragment containing the PPARδ binding element (4 °C/24 h). The PPARδ/DNA binding complex was washed and then incubated with primary PPARδ-specific antibody (room temperature/1 h) and horseradish peroxidase (HRP)-conjugated secondary antibody (room temperature/1 h), and finally analyzed by spectrophotometry (450 nM).

### 4.7. Propidium Iodide (PI)/Annexin V-Fluorescein Isothiocyanate (FITC) Double Stain

To analyze the cell death of human MG63 osteosarcoma cells, PI/annexin v-FITC double stain (V13242, Invitrogen, Carlsbad, CA, USA) was used to monitor the cell apoptosis and necrosis. Briefly, after treatment, cells were collected in cold phosphate-buffered saline. Cell suspension was added into the PI and annexin v-FITC solution and then incubated at room temperature for 15 min. After incubation, the stained cells were analyzed by flow cytometry (BD FACSCanto II, BD Bioscience Inc., San Jose, CA, USA). The percentage of cell apoptosis level was analyzed in CellQuest (BD Bioscience Inc., San Jose, CA, USA).

### 4.8. Statistical Analysis

All assays were performed by 3 repetitions from individual samples. Results were analyzed and presented by mean ± standard error of the mean (SEM). Statistical analysis was calculated by the independent Student’s t-test for two groups of data and analysis of variance (ANOVA) followed by Scheffe’s test for multiple comparisons. A *p*-value < 0.05 was defined as significant.

## Supplementary Materials

Supplementary materials can be found at https://www.mdpi.com/1422-0067/21/13/4720/s1. Figures S1–S3: Gel images of Western blot, as shown in Figure 1, Figure 2 and Figure 3.
