# Supplementary material for: Stearoyl-CoA Desaturase-1 Attenuates the High Shear Force Damage Effect on Human MG63 Osteosarcoma Cells
Source: Int J Mol Sci. 2020 Jul 2;21(13):4720. doi: 10.3390/ijms21134720 (PMC7369751; doi:10.3390/ijms21134720)
Supplement: Supplementary file 1 [file ijms-21-04720-s001.pdf]

Fig. 1A-up-1st

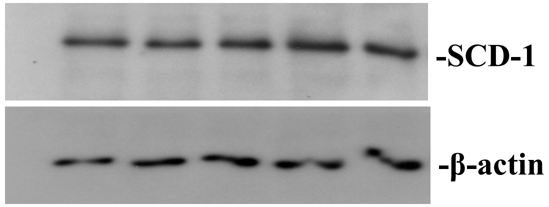

Fig. 1A-down-1st

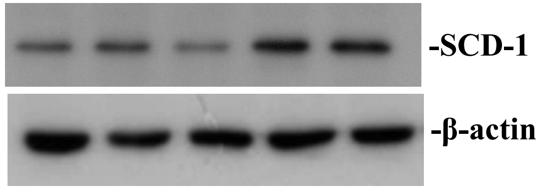

Fig. 1A-up-2nd

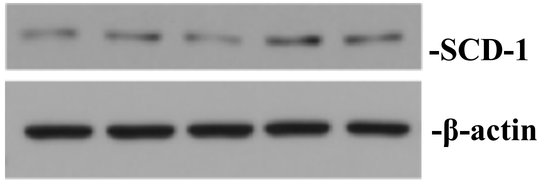

Fig. 1A-down-2nd

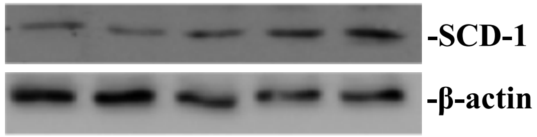

Fig. 1A-up-3rd

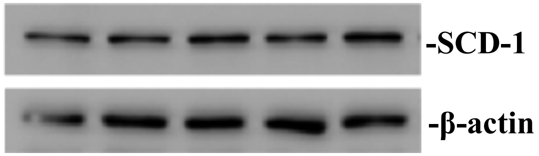

Fig. 1A-down-3rd

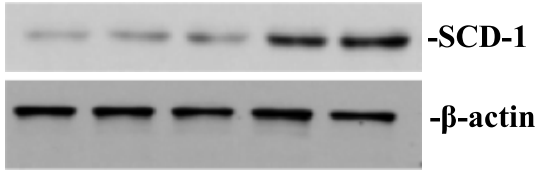

Figure S1: Gel images of Western blot in Figure 1A.

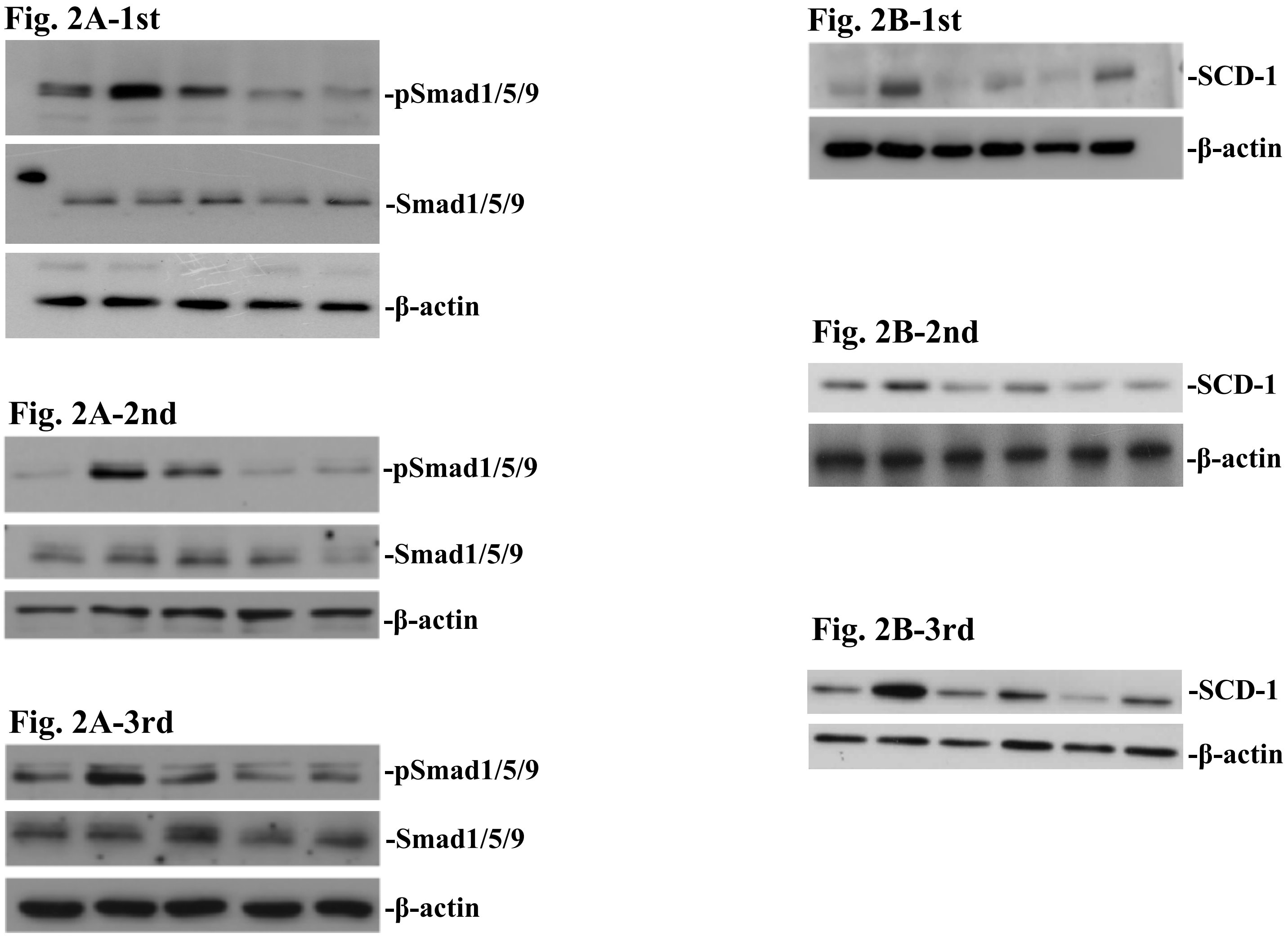

Figure S2: Gel images of Western blot in Figure 2A-B.

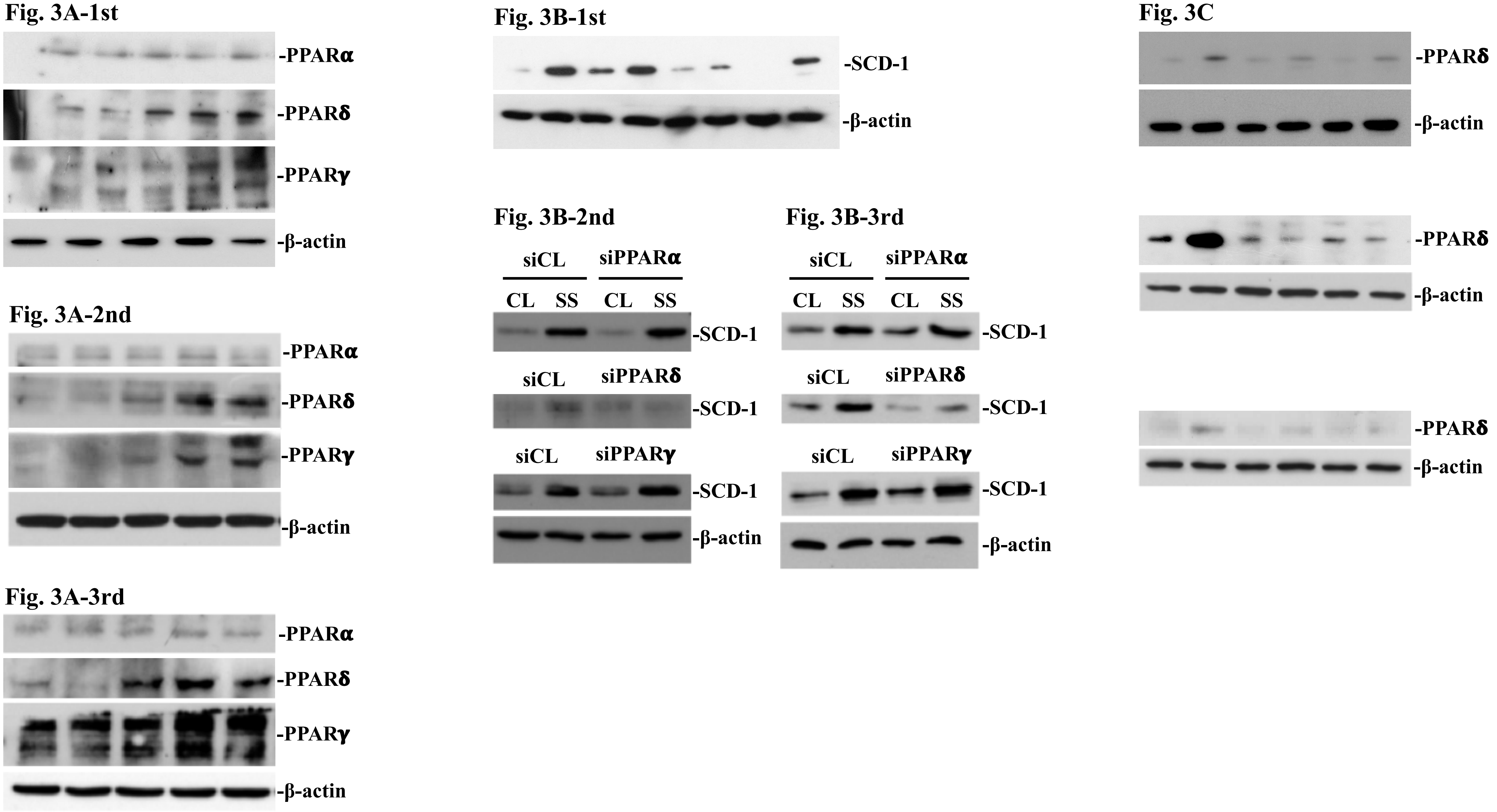

Figure S3: Gel images of Western blot in Figure 3A-C.
